# Analysis of the indicators of the Family Health Program in the Metropolitan Region of São Paulo

**DOI:** 10.1590/S1679-45082018GS4174

**Published:** 2018-08-01

**Authors:** Paulo Augusto Monteclaro Cesar, Márcia Mello Costa De Liberal, Valdecir Marvulle, Paola Zucchi

**Affiliations:** 1 Universidade Federal de São Paulo, São Paulo, SP, Brazil.; 2 Universidade Federal do Oeste da Bahia, Barreiras, BA, Brazil.; 3 Universidade Federal do ABC, Santo André, SP, Brazil.

**Keywords:** Family health strategy, Health, Family, Primary health care, Estratégia saúde da família, Saúde, Família, Atenção primária à saúde

## Abstract

**Objective:**

To analyze the Family Health Program replaced by the Family Health Strategy in 2011, based on health indicators and diseases classified as primary care sensitive.

**Methods:**

This was a descriptive, analytical and documental study carried out in the Metropolitan Region of São Paulo between 2002 and 2007. We analyzed data from Health observatory for the Metropolitan Region of São Paulo. Pearson’s correlation and the Statistical Package for the Social Sciences software version 17.0 were used to calculate data associations.

**Results:**

We used 30 of the 31 health indicators of 24 from the 39 studied municipalities. A total of 720 (100%) health primary care sensitive indicators were analyzed in the Metropolitan Region of São Paulo.

**Conclusion:**

Percentages of improvements and worsening were low. In addition, some data were not presented. The majority of indicators remained stable.

## RESUMO

**Objetivo:**

Analisar o Programa Saúde da Família, substituído pela Estratégia Saúde da Família em 2011, considerando os indicadores de saúde e as doenças classificadas como sensíveis à Atenção Primária.

**Métodos:**

Trata-se de estudo descritivo, analítico e documental, realizado na Região Metropolitana de São Paulo, no período de 2002 a 2007. Foram analisados dados fornecidos pelo Observatório de Saúde da Região Metropolitana de São Paulo. Após a coleta, calcularam-se, com base na correlação de Pearson e por meio do *software* SPSS, versão 17.0, as associações entre os dados.

**Resultados:**

Foram usados 30 dos 31 indicadores de saúde de 24 dos 39 municípios pesquisados. Foram analisados 720 (100%) indicadores de saúde sensíveis à Atenção Primária na Região Metropolitana de São Paulo.

**Conclusão:**

Foram baixos os percentuais de melhoras e pioras, ou, ainda, os dados não foram apresentados. A maioria permaneceu estável.

## INTRODUCTION

In the end of 1970s, the World Health Organization (WHO) proposed developing countries to create and apply an action program geared for health improvement in primary care. The concept mark of these activities was elaborated in Declaration of Alma-Ata of 1978.^1^ It is important to highlight that, from this concept, Brazil has guided its public health policies directed to primary care actions in 1996 implemented in the Unified Health System (UHS - *Sistema Único de Saúde*) and Family Health Program (FHP) as national policies of primary care to provide better health care for population.^2^

Primary care sensitive indicators (PCSI) express a set of health problems in which effective primary care would reduce the risk of hospitalization. Activities as prevention, diagnosis, and treatment of primary care diseases reduce the number of hospitalizations. Therefore, at places where hospitalization rate is high due to primary care sensible conditions, the primary care service is deficient or has low coverage.^3^

Caminal-Homar et al.,^4^ described the use of PCSI in Spain and concluded that 75% to 85% of population requires only primary care and 10% of them need more specialized care. They also reported that hospitalizations because of primary care sensible conditions are indirect measures of effective health.

Bermúdez-Tamayo et al.,^5^ pointed out that factors that should be considered for PCSI analysis, such as hospitalization in different regions, municipalities characteristics, differences of results between adult and child population, municipalities with same characteristics and small number of municipalities used in some studies that would lead to low statistical power.

Based on these premises, guidelines of the program involved promotion of diseases prevention, health, and also, rehabilitation. The fact that these actions are in constant transformation for updating and promote further development, was the driven action that made the so-called FHP, which was implemented in Brazil by the Ministry of Health in 1994, to be replaced in 2011 to Family Health Strategy (FHS).^6^ The FHS requires a greater number of professionals and sites compared with traditional health units, therefore require higher finance investment, considering that this new model of delivery care is in constant development and covers a large portion of the national territory.^7^

Such changes in how to delivery health care services are need to be analyzed and evaluated, such as in both qualitative and quantitative manner in order to enable observation of significant improvement in health population. In 2008, considering this evaluation, the Ministry of Health approved an indicator health list,^8^ which served as tool for primary care assessment. This list contains a variety of diseases that requires patients hospitalization and might be influenced by primary care actions.

The Ministry of Health defines as strategic goal a better primary care service delivery. In national context, we already evaluated the FHS and PCSI, however, using as variables only for hospitalization because of primary care sensible conditions or basic indicators^7,8^

## OBJECTIVE

To verify whether Family Health Strategy Program improves health conditions of populations considering health indicators and diseases classified as primary care sensitive.

## METHODS

This was a documental, descriptive, analytical, and retrospective study. The ethical and research committee of the *Universidade Federal de São Paulo* approved in the study, nº 0052/12HE.

The research analyzed the PCSI and its relationship with FHS covering in the Metropolitan Region of São Paulo (MASP) distributed in seven regions and including 39 municipalities. The study was carried out from 2002 to 2007 and units were chosen after FHS implementation in many municipalities of the region. We excluded from analysis municipalities that, in this period, had partially, did not have or discontinued FHS programs. The choosing of MASP was justified in order to cover a large number of inhabitants due to great number of municipalities that offered FHS, and also because the region has an electronic and specific data source, the Health observatory for the Metropolitan Region of São Paulo,^9^ which allows a depth analysis of results of the strategy by using the PCSI.

The analyzed variables included percentage of population covered by the FHS and PCSI subdivided in primary care indicators, number of hospitalizations by primary care sensitive diseases. This group is made up by primary care indicators, that is, the diseases in which care effectiveness reduces the number of hospitalizations. To enable analysis of FHS evolution covering municipalities the correlations were calculated among percentages of population assisted by the program in each year in the analyzed period.

Based on this data, municipalities were divided into three different groups: those with (1) increase; (2) decrease, and (3) stagnation of proportion of population covered throughout the time. In the sequence, we analyzed PCSI and its relation with FHS covering.

For this reason, we considered what occurred with indicators in each one of three groups of municipalities previous described. The level of significance adopted for Pearson’s correlation was p<0.05. The Statistical Package for the Social Sciences (SPSS), version 17.0, was used for calculations.

## RESULTS

Of 39 municipalities of MASP, 24 (61.5%) were included in the study. The assessment of progress of FHS covering, 12 (50%) of municipalities comprised the group in which there was increase, 9 (37.5%) composed the group with FHS covering that remained stable, and in 3 (12.5%) municipalities the covering was reduced.

In the group that an increase in covering was observed, a total of 30 PCSI were analyzed in each one of the 12 municipalities, totalizing 360 (100%) indicators. Of these, 40 (11.1%) had improvement, 268 (74.4%) remained stable, and 38 (10.2%) worsened, in addition 14 (3.9%) municipalities without data. When analyzed the mean, 3.3 had improvement, 22.5 remained stable, 3.2 worsened, and 3.9 did not have data (Table 1).

**Table 1:** Situation of Primary Care Sensitive Indicators from 2002 to 2007 in Municipalities of Metropolitan Region of São Paulo

PCSI				

Regions and municipalities	Improved n (%)	Remained unchanged n (%)	Worsened n (%)	No data n (%)
Itapevi	4 (13.3)	24 (80.0)	2 (6.6)	0 (0)
Taboão da Serra	0 (0)	24 (80.0)	4 (13.3)	2 (6.6)
São Caetano do Sul	0 (0)	21 (70.0)	7 (23.3)	2 (6.6)
Santo André	2 (6.6)	23 (76.6)	4 (13.3)	1 (3.3)
Diadema	4 (13.3)	25 (83.0)	1 (3.3)	0 (0)
Cajamar	2 (6.6)	25 (83.3)	1 (3.3)	2 (6.6)
Santa Isabel	8 (26.6)	21 (70.0)	1 (3.3)	0 (0)
Poá	5 (16.6)	21 (70.0)	2 (6.6)	2 (6.6)
Itaquaquecetuba	7 (23.3)	22 (73.3)	0 (0)	1 (3.3)
Biritiba Mirim	1 (3.3)	25 (83.3)	0 (0)	4 (13.3)
São Paulo	5 (16.6)	16 (53.3)	9 (30.0)	0 (0)
Guarulhos	2 (6.6)	21 (70.0)	7 (23.0)	0 (0)
Total	40 (11.1)	268 (74.4)	38 (10.2)	14 (3.9)
Mean	3.3 (11.0)	22.5 (75.0)	3.2 (10.7)	1.0 (3.3)

PCSI: Primary Care Sensitive Indicators.

Source: Brasil. Ministério da Saúde. Organização Pan-Americana da Saúde. Salas de situação em saúde: compartilhando as experiências do Brasil. Organizadores José Moya, João Baptista Risi Junior, Ayrton Martinello, Ernani Bandarra, Helvécio Bueno, Otaliba Libânio de Morais Neto [Internet]. Brasília (DF): Ministério da Saúde, Organização Pan-Americana da Saúde; 2010 [citado 2018 Jan 5]. Disponível em: http://www.paho.org/bra/index.php?option=com_docman&view=download&category_slug=informacao-e-analise-saude-096&alias=958-salas-situacao-em-saude-compartilhando-as-experienciasdo-brasil-8&Itemid=965^(9)^

In the group that covering remained stable, we analyzed 30 PCSI, and each one of the nine municipalities, totalizing 270 (100%) indicators. Of these, 15 (5.5%) had improvement, 229 (84.8%) remained stable and 9 (3.3%) worsened, while 17 (6.2%) did not have data. When analyzed the mean values, we observed 1.7 indicator had improvement, 25.4 remained stable, and 1 worsened and 1.9 did not have data (Table 2).

**Table 2:** Situation of Primary Care Sensitive Indicators from 2002 to 2007 in Municipalities of Metropolitan Region of São Paulo

PCSI				

Regions and municipalities	Improved n (%)	Remained unchanged n (%)	Worsened n (%)	No data n (%)
Pirapora do Bom Jesus	3 (10.0)	24 (80.0)	0 (0)	3 (10.0)
Jandira	2 (6.6)	26 (86.0)	0 (0)	2 (6.6)
Santana do Parnaíba	0 (0)	26 (86.0)	2 (6.6)	2 (6.6)
Juquitiba	5 (26.6)	22 (73.3)	1 (3.3)	2 (6.6)
São Lourenço da Serra	0 (0)	26 (86.9)	0 (0)	4 (13.3)
São Bernardo do Campo	1 (3.3)	27 (90.0)	1 (3.3)	1 (3.3)
Franco da Rocha	2 (6.6)	25 (83.0)	2 (6.6)	1 (3.3)
Mairiporã	1 (3.3)	26 (86.0)	2 (6.6)	1 (3.3)
Ferraz de Vasconcelos	1 (3.3)	27 (90.0)	1 (3.3)	1 (3.3)
Total	15 (5.5)	229 (84.8)	9 (3.3)	17 (6.2)
Mean	1.7 (5.7)	25.4 (84.7)	1 (3.3)	1.9 (6.3)

PCSI: Primary Care Sensitive Indicators.

Source: Brasil. Ministério da Saúde. Organização Pan-Americana da Saúde. Salas de situação em saúde: compartilhando as experiências do Brasil. Organizadores José Moya, João Baptista Risi Junior, Ayrton Martinello, Ernani Bandarra, Helvécio Bueno, Otaliba Libânio de Morais Neto [Internet]. Brasília (DF): Ministério da Saúde, Organização Pan-Americana da Saúde; 2010 [citado 2018 Jan 5]. Disponível em: http://www.paho.org/bra/index.php?option=com_docman&view=download&category_slug=informacao-e-analise-saude-096&alias=958-salas-situacao-em-saude-compartilhando-as-experienciasdo-brasil-8&Itemid=965^(9)^

For each of the three municipalities in which we analyzed 30 PCSI, therefore totalizing 90 (100%) indicators. Of these, 2 (22.2%) had improvement, 83 (92.2%) remained stable, 9 (3.3%) worsened and 2 (2.2%) did not have data. In the analysis of general mean of indicators, 0.7 had improvement, 27.6 remained stable, 1 worsened, while 0.7 did not have data (Table 3).

**Table 3:** Situation of Primary Care Sensitive Indicators from 2002 to 2007 in Municipalities of Metropolitan Region of São Paulo

PCSI				

Regions and municipalities	Improved n (%)	Remained unchanged n (%)	Worsened n (%)	No data n (%)
Itapecerica da Serra	0 (0)	28 (93.3)	0 (0)	2 (6.6)
Mauá	1 (3.3)	26 (86.0)	3 (10.0)	0 (0)
Francisco Morato	1 (3.3)	29 (96.6)	0 (0)	0 (0)
Total	2 (2.2)	83 (92.2)	3 (3.3)	2 (2.2)
Mean	0.7 (2.3)	27.6 (92.0)	1 (3.3)	0.7 (2.3)

PCSI: Primary Care Sensitive Indicators.

Source: Brasil. Ministério da Saúde. Organização Pan-Americana da Saúde. Salas de situação em saúde: compartilhando as experiências do Brasil. Organizadores José Moya, João Baptista Risi Junior, Ayrton Martinello, Ernani Bandarra, Helvécio Bueno, Otaliba Libânio de Morais Neto [Internet]. Brasília (DF): Ministério da Saúde, Organização Pan-Americana da Saúde; 2010 [citado 2018 Jan 5]. Disponível em: http://www.paho.org/bra/index.php?option=com_docman&view=download&category_slug=informacao-e-analise-saude-096&alias=958-salas-situacao-em-saude-compartilhando-as-experienciasdo-brasil-8&Itemid=965^(9)^

In general, three groups made up of 24 municipalities, a total of 720 indicators (100%) were analyzed, and 57 of them (7.9%) had improvements, 580 (80.5%) remained stable, 50 (6.9%) worsened and 33 (4.5%) did not have data (Table 4).

**Table 4:** Situation of Primary Care Sensitive Indicators from 2002 to 2007 in Municipalities of Metropolitan Region of São Paulo

PCSI				

Correlated municipalities group with health indicators	Improved n (%)	Remained unchanged n (%)	Worsened n (%)	No data n (%)
Municipalities group in which FHS covering increased	40 (11.1)	268 (74.4)	38 (10.2)	14 (3.9)
Municipalities group in which FHS covering remained stable	15 (5.5)	229 (84.8)	9 (3.3)	17 (6.2)
Municipalities group in which FHS covering decreased	2 (2.2)	83 (92.2)	3 (3.3)	2 (2.2)
Total	57 (7.9)	580 (80.5)	50 (6.9)	33 (4.5)

FHS: Family Health Strategy; PCSI: Primary Care Sensitive Indicators.

Source: Brasil. Ministério da Saúde. Organização Pan-Americana da Saúde. Salas de situação em saúde: compartilhando as experiências do Brasil. Organizadores José Moya, João Baptista Risi Junior, Ayrton Martinello, Ernani Bandarra, Helvécio Bueno, Otaliba Libânio de Morais Neto [Internet]. Brasília (DF): Ministério da Saúde, Organização Pan-Americana da Saúde; 2010 [citado 2018 Jan 5]. Disponível em: http://www.paho.org/bra/index.php?option=com_docman&view=download&category_slug=informacao-e-analise-saude-096&alias=958-salas-situacao-em-saude-compartilhando-as-experienciasdo-brasil-8&Itemid=965^(9)^

In MASP, from 2002 to 2007, most (80.5%) of PCSI remained stable, and FHS covering, within the period, was not directed related with indicators that improved.

The indexes proportion that improved in the municipalities group which FHS covering increased was significantly higher than those which covering remained stable or reduced. However, the indexes proportion that remained unchanged in the municipalities group that covering increased was significantly lower than in those which remained stable or presented reduction. Indexes proportion that worsened in the municipalities group in which covering increased was significantly higher than in those in which it remained stable or decreased.

In municipalities that FHS covering remained unchanged, indicators did not differ from those municipalities that covering had decreased. However, in the group that covering increased, there was higher indexes proportion that improved and indexes that worsened. These results enabled to affirm that increase in FHS covering was not directly related with PCSI improvement. In municipalities that there was increase in FHS covering, a higher proportion of indicators improved compared with other municipalities.

## DISCUSSION

Other studies, with similar goals, such as the Health observatory for the Metropolitan Region of São Paulo study,^9^which evaluated hospitalization for primary care sensible conditions from 2000 to 2010, showed increase in admission rate because of primary care sensible conditions of MASP in a specific timeframe.

Our data also show that relationship between increase of FHS covering and drop in hospitalization rates because of primary care sensible conditions were not uniform.

Boing et al.,^10^ evaluated the number of hospitalizations associated with primary care sensible conditions from 1998 to 2009, and observed an annual mean reduction of 3.7% and the São Paulo State was not among those states that had greater reduction in number of hospitalizations. Authors also used PCSI association and observed a reduction in number of hospitalizations in most of units, although some of them revealed stability or increase of hospitalization because of primary care sensible conditions. Factors such as socioeconomic conditions and private or specialized health service are related with hospitalization rates by primary care sensible conditions, and they may generate regional differences. They still affirm that in no state the number of hospitalization by primary care sensible conditions increased in the studied period. However, data analyzed from 2002 to 2007 revealed that number of hospitalization had increased. Rehem et al.,^11^ described hospitalization because of primary care sensible conditions in the State of São Paulo, considering the years 2000 and 2007. However, there was reduction of 435,594 in 2000 to 429,070 in 2007. However, they reported increase from 40.75% in number of hospitalization in São Paulo, and they also observed improvement in other regions related with enlargement of FHS in the State, and the increase in hospitalization did not occur homogenously, but a great difference among regional health departments was seen.

In 2008, Torres et al.,^12^ studied hospitalizations because of primary care sensible conditions in hospitals of the district of São Paulo, and showed that rise in number of hospitalization because of urinary tract infections, bacterial pneumonia, and blood hypertension. These data pointed out rise in number of hospitalizations similar to the present study, although this rise occurred only in district of São Paulo and in the year of 2008.

Ferreira et al.,^13^ did a work in Ribeirão Preto (SP) and they reported a reduction of 9.6% in number of hospitalization for sensible conditions between 2000 and 2007. These results are different from our study, perhaps, because this was a comparative study among hospitalizations because of primary care sensible diseases in 2000 and 2007, and no evolution of the disease was observed in the period of hospitalizations. However, the existence of limitations of FHS need to be considered because of increase in number of hospitalization in groups with important problems.

Dias-da-Costa et al.,^14^ studied quality of primary care considering hospitalization by sensible conditions and this type of care in Pelotas (RS) from 1995 to 2004, and they observed a reduction in number of hospitalizations. However, their study did not use the complete list of diseases adopted by the Ministry of Health. Nedel et al.,^15^ evaluated FHS by incidence of primary care sensible conditions in Bagé (RS) and they pointed out that, in a sample including 1,200 patients who were hospitalized between September 2006 and January 2007 in the municipality, 42.6% were admitted because of primary care sensible conditions. They conclusion highlighted that, in some hospitalized groups that adopted the FHS program, the number of hospitalization was lower than in those in which traditional primary care was adopted.

Rodrigues-Bastos et al.,^16^ analyzed hospitalizations associated with primary care sensible conditions in Juiz de Fora (MG) between 2002 and 2005, and 2006 and 2009, by comparing results in these years. Hospitalization rates were 7.74 per 1,000 inhabitants from 2002 to 2005, growing to 8.81 per 1,000 inhabitants between 2006 and 2009. Pazó et al.,^17^ studied hospitalization associated with primary care sensible conditions in Espirito Santo from 2005 to 2009 which represented 28.5% of the total in 2005, and 23.2% in 2009. The number hospitalization reduced, although irregular, only for primary care sensible conditions.

In the study adopted as reference for this study authors used the sum of hospitalizations by primary care sensible diseases and to PCSI.^18^ It was also possible to observe that the period defined in the study showed lack of enough data for more precisely analysis of results of actions in health area. We also observed the importance of emphasizing that FHS covering in MASP in 2002 was, in the beginning, made up by 17.4% of population and became 33.7% in 2007, therefore showing a significant increase (almost double).

In relation to hospitalization due to primary care sensible conditions, Nedel et al.,^19^ argued that these indicators must be analyzed based on sensibility and specificity, and not in frequency of observed disease. These indicators should be also used in combinations with socioeconomic environment and their effects on the social and demographic structure because they depend on organization of health services, its availability, access and adherence to technological model of current primary care.

We observed that 12 (50%) municipalities had an increase of population assisted by the FHS. Of them, 9 (37%) the FHS covering remained unchanged and, in 3 (12.5%) there was a decrease in covering between 2002 and 2007. In the analysis of relationship between PCSI and FHS covering in MASP from 2002 to 2007, 57 (7.9%) of indicators improved, whereas 580 (80.5%) remained unchanged and 50 (6.9%) decreased.

Only half of municipalities that served as sample for this study regarding FHS covering had a mild increase. In 9 municipalities, conditions remained unchanged. However, in relation to correlation analysis between PCSI and FHS covering, only a small portion registered improvements, whereas the majority (80.5%) remained unchanged in relation to socioeconomic dynamic of this region during the period of 5 years (2002 – 2007). These findings show that efforts done by public and private spheres to improve general numbers did not result, in practice, in more efficient policy in the health care.

It was also possible to verify a higher proportion of indicators that worsened throughout the analyzed period, therefore showing a direct correlation between indicators that improved with increase of FHS covering are still not established in clear and effective manner. Reason for this behavior can be explained because of FHS became recognized primary care policies in 1996.

## CONCLUSION

Based on analysis of the Family Health Program, which in 2011 started to be known as the Family Health Strategy, we identified considering percentage of health indicators and diseases classified as sensible to primary care that, after almost two decades of activities, this family care based model is under expansion in Brazil. The proof that this program is under growing is the small improvement and worsening of indexes, or, even, the lack of data to provide a robust assessment. In addition, we observed that most of indicators remained stable.

Still, the assessment period seemed insufficient to obtain accurate conclusions to show and identify the real need of more efforts by the sanitary authorities given the increase in availability of the service delivered that is vital to the development of health care infrastructure in the Brazilian society.
